# Retinal features as predictive indicators for high myopia: insights from explainable multi-machine learning models

**DOI:** 10.3389/fbioe.2025.1609639

**Published:** 2025-10-13

**Authors:** Haohan Zou, Jing Liu, Shenda Shi, Saiguang Ling, Qian Fan, Yan Huo, Zhou Dong, Guoge Han, Shengjin Wang, Yan Wang

**Affiliations:** ^1^ Tianjin Eye Hospital, Tianjin, China; ^2^ Tianjin Key Lab of Ophthalmology and Visual Science, Tianjin Eye Institute, Tianjin, China; ^3^ Nankai University Eye Institute, Nankai University, Tianjin, China; ^4^ Department of Ophthalmology, Beijing Friendship Hospital, Capital Medical University, Beijing, China; ^5^ HuaHui Jian AI Tech Ltd., Tianjin, China; ^6^ EVision technology (Beijing) Co., Ltd., Beijing, China; ^7^ Department of Electronic Engineering, Tsinghua University, Beijing, China

**Keywords:** high myopia, retinal imaging omics, machine learning, deep learning, shapley additive exPlanation

## Abstract

**Objectives:**

To investigate the role of retinal characteristics for high myopia (HM) prediction based on multi-machine learning (ML) and to provide an interpretable framework for the results.

**Methods:**

A total of 2981 patients (2981 eyes) were included, comprising 1191 HM eyes and 1790 non-HM eyes. A deep semantic segmentation network was used to quantify retinal structural parameters. Five ML algorithms were evaluated to develop predictive models, and SHapley Additive exPlanations method was applied to analyze feature contribution to the outcomes.

**Results:**

The eXtreme Gradient Boosting achieved an accuracy of 0.81 (95% confidence interval [CI] 0.78–0.85), and an area under the receiver operating characteristic curve of 0.87 (95% CI 0.84–0.89), outperforming other models. The 12 most important factors affecting prediction included tessellation density, seven vascular parameters, two parapapillary atrophy parameters, and two optic disc parameters. The tessellated density >0.025, width of parapapillary atrophy >400 um, parapapillary atrophy area >0.60 × 10^6^ um^2^ were associated with an increased risk of HM. The mean curvature of the arteries >0.00063, diameter of vessels >55.2 um, curvature of the veins >0.00128, vertical diameter of the optic disc >1320 um, diameter of veins >58.5 um, and diameter of artery >47.1 um was associated with a decreased risk of HM.

**Conclusion:**

The XGBoost model outperformed other algorithms, and SHAP-derived cut-off values for critical risk factors enhanced clinical interpretability.

## 1 Introduction

The global prevalence of high myopia (HM) has risen significantly, drawing attention due to its association with increased risks of blindness and substantial social burdens (Baird et al., 2020; Xu et al., 2021; Shinojima et al., 2022). Patients with HM are more than 50 percent more likely to develop cataracts, glaucoma, macular degeneration, retinal detachment, and optic neuropathy (Modjtahedi et al., 2021; Verhoeven et al., 2015). These risks increase further with age, refractive error, and axial length (Haarman et al., 2020). Thus, early detection and diagnosis of HM is crucial.

HM often results in alterations to the fundus structures, which may not be evident in the early stages (Salih et al., 2023). As a non-invasive and straightforward diagnostic tool, retinal imaging provides comprehensive information about anatomical structures and optical characteristics (Li et al., 2021; Chen et al., 2024). However, traditional image analysis methods, such as meta-analyses of pathologic myopia (META-PM) and the atrophy, traction, and neovascularization classification systems (Ohno-Matsui et al., 2016; Ruiz-Medrano et al., 2019), are commonly dependent on the knowledge of ophthalmologists. These methods frequently fail to provide rapid and precise outcomes, and the requisite expertise limits their broader applicability. Hence, automatic identification and quantification of key features, and accurate data interpretation is vital for the diagnosis and prediction of disease progression (Conti et al., 2021). Recent advancements in computer technology and machine learning (ML) algorithms have made radiomics invaluable in clinical decision-making (Sun R. et al., 2018; Sun et al., 2021; Yu et al., 2021). By combining retinal imaging with omics, radiomics can automatically extract data on fundus shape, texture, geometric characteristics, and relative positions (Zhang et al., 2025). This approach enables rapid identification of HM features and provides valuable insights.

This study investigated the role of fundus features in predicting HM using a retinal imaging omics-based analytical method. Additionally, SHapley Additive exPlantations (SHAP) was employed to help ophthalmologists better understand the relationships between these features and HM, thereby supporting accurate screening and prediction of high-risk groups.

## 2 Methods

### 2.1 Study approval

This study was registered with the Chinese Clinical Trial Register (ChiCTR2100049885) and approved by the Ethics Committee of Tianjin Eye Hospital (TJYYLL2021018) in accordance with the Declaration of Helsinki. The study followed the Strengthening the Reporting of Observational Studies in Epidemiology (STROBE) guidelines (Von Elm et al., 2007). The ethics committee waived the requirement for informed consent because the data were de-identified before the study to ensure patient privacy.

### 2.2 Patients and data

The examination records of 4,048 patients from August 2019 to December 2021 were retrospectively collected using an electronic medical record system. Of these, 53.9% (n = 2,183) were male and 46.1% (n = 1,865) were female. The mean age was 22.72 ± 5.24 (range 16–45) years. The inclusion criteria included age ≥16 years, diagnosis of refractive error, and a best-corrected visual acuity of at least 0.0 (LogMAR). Patients with disease that affect vision acuity, such as cataract, various types of glaucoma, retinal tears, retinal detachment, macular schisis, macular holes, epiretinal membranes, macular neovascularization, were excluded. Patients with systemic diseases such as hypertension, diabetes, and cardiovascular diseases that affected fundus characteristics were excluded. To avoid inter-eye effects, only the left side of the eye was selected for analysis.

In order to obtained the accuracy refraction, all patients underwent both subjective and objective refraction measurements conducted before and after cycloplegia. Spherical power ranged from −0.25 D to −13.00 D, and cylinder power ranged from −0.50 D to −6.00 D. The spherical equivalent (SE) of the refractive error was calculated as spherical error +1/2 cylindrical error. The subjects included in this study were all cases of simple myopia. Data were divided into two groups: the non-HM group (SE > −6.00 D and ≤ −0.50 D) and the HM group (SE ≤ −6.00 D) (Jong et al., 2021). Fundus images were taken using an automated image quality control system, with images centered on the macula and always included the optic disc.

### 2.3 Feature recognition and quantification

Object detection and semantic segmentation algorithms were used to extract and quantify retinal features, including quantitative information on tessellation, optic discs, optic cups, parapapillary atrophy, and retinal vascular structures. The annotation was performed using a semi-automatic machine-assisted workflow. Two board-certified ophthalmologists (specializing in fundus diseases with ≥5 years of experience) independently validated the annotations. The first physician performed initial corrections, and the second (with ≥8 years of experience) served as adjudicator. Given the second reviewer’s greater clinical experience, their annotations were designated as ground truth. The methodology details are provided in Document S1.

### 2.4 Model construction and evaluation

Data from the baseline examinations were incorporated into a ML modeling. Five ML algorithms (eXtreme Gradient Boosting (XGBoost), Categorical Boosting (CatBoost), Light Gradient Boosting Machine (LightGBM), Random Forest (RF), and Neural Network (NN)) were utilized to build the model. To mitigate overfitting, the data were randomly split into separate training and testing datasets. Model performance was assessed using 5-fold cross-validation (Document S2). Evaluation metrics included sensitivity, specificity, accuracy, precision, F1 score, and the area under the receiver operating characteristic curve (AUROC). Higher values for these metrics indicate superior model performance. The SHAP method evaluated the importance of each input variable by analyzing the average impact on model output with and without the target input variable. Model training was conducted using Python 3.6 (https://www.python.org).

### 2.5 Statistical analyses

Data were analyzed using SPSS (version 26.0; IBM Corp., Armonk, NY, United States). Outliers were identified and removed using Tukey’s test with a coefficient of 1.5. Categorical data were expressed as frequencies and percentages and compared using the χ^2^ test. The normality of continuous parameters was assessed with the Kolmogorov–Smirnov test, with P > 0.05 indicating a normal distribution. Normally distributed data were expressed as mean ± standard deviation and compared using independent samples *t*-tests. A two-tailed p-value of 0.05 or less was considered statistically significant. To maintain consistency, data from the same eye was selected for analyses to avoid inter-eye variability.

## 3 Results

### 3.1 Basic patient information

**FIGURE 1:**
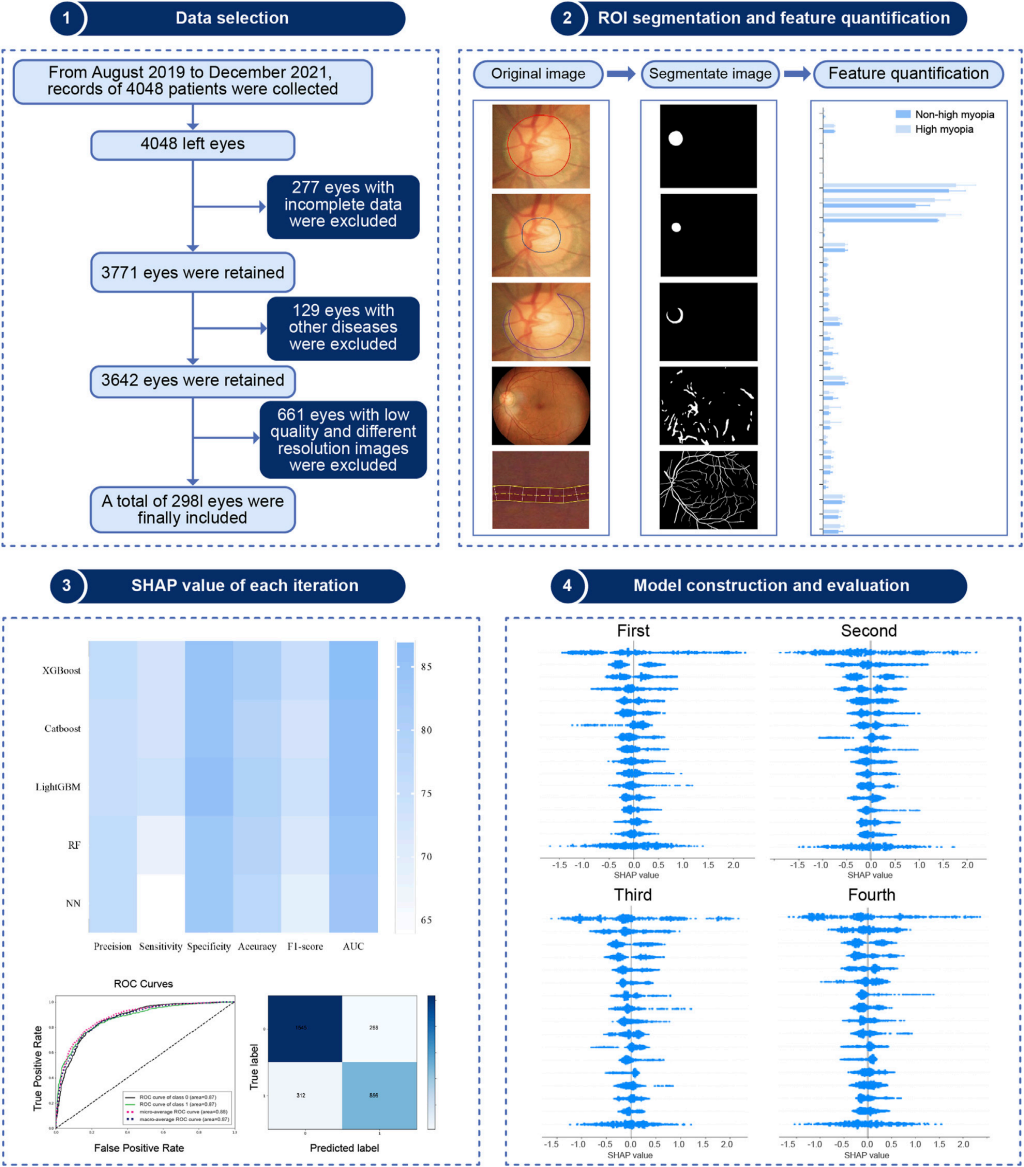
Flow chart for data selection and analyses in the study.

The study followed the steps shown in Figure 1, which included clinical data collection, image segmentation and quantification, feature engineering, model construction and evaluation, and a SHAP analysis.

A total of 2,981 eyes were included. Of these, 1,191 (39.9%) were classified as highly myopic, whereas 1790 (60.1%) were non-highly myopic. The following variables including age, sex, refraction, intraocular pressure, visual acuity, and 29 retinal characteristics were obtained. The baseline analysis of the dataset is shown in Table 1. The average age of 2,981 patients were 22.71 ± 5.24 years, including 1539 males (51.63%) and 1442 females (48.37%).

**TABLE 1:** Baseline characteristics of the participants included in the analysis.

Variables	High myopia (n = 1191)	Non-high myopia (n = 1790)	t/χ^2^ value	*P* value
Age (y)	23.15 ± 5.05	22.29 ± 5.42	−4.26	<0.001
Sex (Male)	646 (54.24%)	893 (49.89%)	5.42	0.02
SE (D)	−8.49 ± 1.35	−3.23 ± 1.18	109.11	<0.001
IOP (mmHg)	16.14 ± 2.33	15.75 ± 2.45	−4.32	<0.001
UCVA (logMAR)	0.16 ± 0.15	0.05 ± 0.05	28.85	<0.001
ODA (mm^2^)	1.08 ± 0.28	1.18 ± 0.27	9.98	<0.001
HOD (mm)	1.05 ± 0.18	1.12 ± 0.17	11.74	<0.001
VOD (mm)	1.32 ± 0.15	1.35 ± 0.14	5.90	<0.001
OCA (mm^2^)	0.22 ± 0.12	0.26 ± 0.12	9.02	<0.001
HOC (mm)	0.50 ± 0.14	0.55 ± 0.14	9.49	<0.001
VOC (mm)	0.55 ± 0.15	0.60 ± 0.15	7.88	<0.001
C/D ration	0.20 ± 0.07	0.22 ± 0.07	7.43	<0.001
HCDR	0.47 ± 0.082	0.49 ± 0.78	5.43	<0.001
VCDR	0.42 ± 0.08	0.44 ± 0.78	7.89	<0.001
PPAA (mm^2^)	0.69 ± 0.33	0.44 ± 0.27	−21.63	<0.001
HPA (mm)	1.50 ± 0.22	1.36 ± 0.22	−15.33	<0.001
WPA (mm)	0.51 ± 0.20	0.34 ± 0.16	−23.76	<0.001
PPA/ODA ratio	0.67 ± 0.35	0.40 ± 0.29	−21.47	<0.001
WPPA/HOD ratio	0.49 ± 0.22	0.32 ± 0.19	−21.06	<0.001
HPPA/VOD ratio	1.15 ± 0.14	1.05 ± 0.14	−17.51	<0.001
WIR (mm)	0.37 ± 0.05	0.36 ± 0.04	−3.23	0.001
WSR (mm)	0.37 ± 0.05	0.36 ± 0.05	−3.39	0.001
WNR (mm)	0.25 ± 0.06	0.26 ± 0.06	2.92	0.003
WTR (mm)	0.28 ± 0.07	0.29 ± 0.06	2.78	0.005
Fractal dimension	1.50 ± 0.20	1.51 ± 0.17	10.42	<0.001
Vessel density	0.09 ± 0.01	0.10 ± 0.01	11.84	<0.001
Vascular tortuosity (×10^–3^)	0.79 ± 0.09	0.84 ± 1.05	12.94	<0.001
Arterial tortuosity (×10^–3^)	0.64 ± 1.00	0.77 ± 1.02	11.71	<0.001
Venous tortuosity (×10^–3^)	0.87 ± 1.11	0.92 ± 1.37	7.60	<0.001
Mean vessel diameter (mm)	0.055 ± 0.00	0.056 ± 0.00	9.26	<0.001
Mean arterial diameter (mm)	0.049 ± 0.00	0.050 ± 0.00	7.74	<0.001
Mean venous diameter (mm)	0.062 ± 0.00	0.063 ± 0.00	6.34	<0.001
A/V ratio	0.79 ± 0.04	0.78 ± 0.05	3.72	<0.001
Tessellated density	0.08 ± 0.06	0.04 ± 0.04	−24.41	<0.001

IOP, intraocular pressure; UCVA, uncorrected visual acuity; ODA, optic disc area; HOD, horizontal diameter of the optic disc; VOD, vertical diameter of the optic disc; OCA, optic cup area; HOC, horizontal diameter of the optic cup; VOC, vertical diameter of the optic cup; C/D ration, cup-to-disc area ratio; HCDR, horizontal cup-to-disc ratio; VCDR, vertical cup-to-disc ratio; PPAA, parapapillary atrophy area; HPA, height of parapapillary atrophy; WPA, width of parapapillary atrophy; PPA/ODA, ratio, parapapillary atrophy-to-optical disc area ratio; WPPA/HOD, ratio, width of parapapillary atrophy-to-horizontal diameter of the optic disc ratio; HPPA/VOD, ratio, height of parapapillary atrophy-to-vertical diameter of the optic disc ratio; WIR, width of inferior rim; WSR, width of superior rim; WNR, width of nasal rim; WTR, width of temporal rim; A/V ratio, arterial-to-venous ratio.

### 3.2 Algorithm evaluation

All participants were divided into five equal subsets, with one subset serving as the validation set and the remaining four subset serving as the training set for 5-fold cross-validation. Table 2 lists the performance metrics for each model. The results showed that XGBoost performed best across the five ML, with an accuracy of 0.81 (95% confidence interval [CI], 0.78–0.85) and an AUROC of 0.87 (95% CI, 0.84–0.89).

**TABLE 2:** Performance of different models in the validation set.

Models	Precision (95% CI)	Sensitivity (95% CI)	Specificity (95% CI)	Accuracy (95% CI)	F1-score (95% CI)	AUC (95% CI)
XGBoost	0.76 (0.73–0.80)	0.73 (0.71–0.76)	0.85 (0.81–0.88)	0.81 (0.78–0.85)	0.75 (0.73–0.78)	0.87 (0.84–0.89)
Catboost	0.75 (0.74–0.78)	0.73 (0.70–0.75)	0.85 (0.81–0.88)	0.79 (0.77–0.81)	0.73 (0.70–0.77)	0.86 (0.82–0.88)
LightGBM	0.75 (0.72–0.77)	0.74 (0.71–0.77)	0.86 (0.82–0.89)	0.80 (0.79–0.84)	0.74 (0.71–0.79)	0.86 (0.83–0.90)
RF	0.76 (0.74–0.79)	0.68 (0.65–0.71)	0.83 (0.80–0.84)	0.79 (0.75–0.83)	0.72 (0.69–0.73)	0.85 (0.82–0.88)
NN	0.76 (0.72–0.81)	0.64 (0.60–0.67)	0.83 (0.80–0.87)	0.78 (0.72–0.83)	0.69 (0.64–0.75)	0.84 (0.79–0.88)

AUC, area under the curve; CI, confidence interval.

**FIGURE 2:**
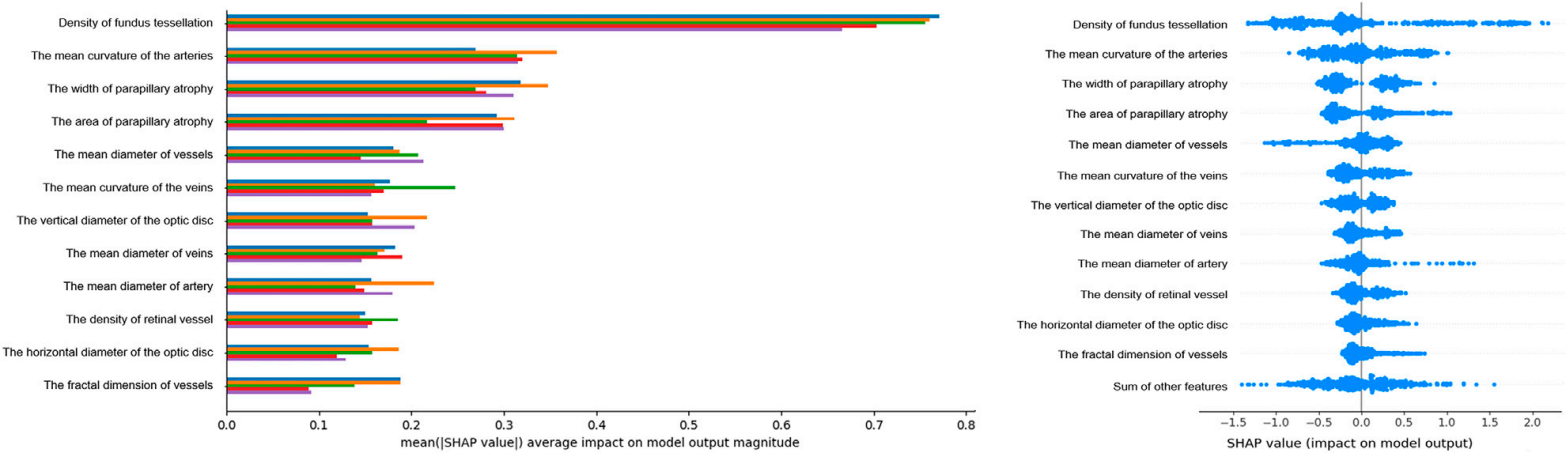
Feature Importance Visualization and SHAP Contributions. Left, Feature importance plot of the XGBoost model: this plot highlights the top 16 features influencing HM, identified through cross-validation. Features are ranked by their impact. The y-axis shows the average absolute SHAP values. Importance decreases from top to bottom. Right, SHAP summary plot: each row represents a feature, and each point corresponds to a sample. A wider scatter indicates a stronger effect, whereas a distribution near 0 suggests minimal influence on most samples.

Feature importance in the XGBoost model was assessed using feature importance bars based on average absolute SHAP values (Figure 2A). The larger absolute values represented significant influence. We summarized the top 12 features that contributed most significantly to the model predictions. Retinal tessellation density was at the top of the ranking list, followed by the mean arterial curvature, parapapillary atrophy width and area, mean vessel diameter, mean venous curvature, vertical optic disc diameters, mean venous and arterial diameters, retinal vessel density, horizontal optic disc diameters, and vessel fractal dimension. Each point corresponding to the SHAP summary plot (Figure 2B) represents the contribution of each feature of each patient to the model.

**FIGURE 3:**
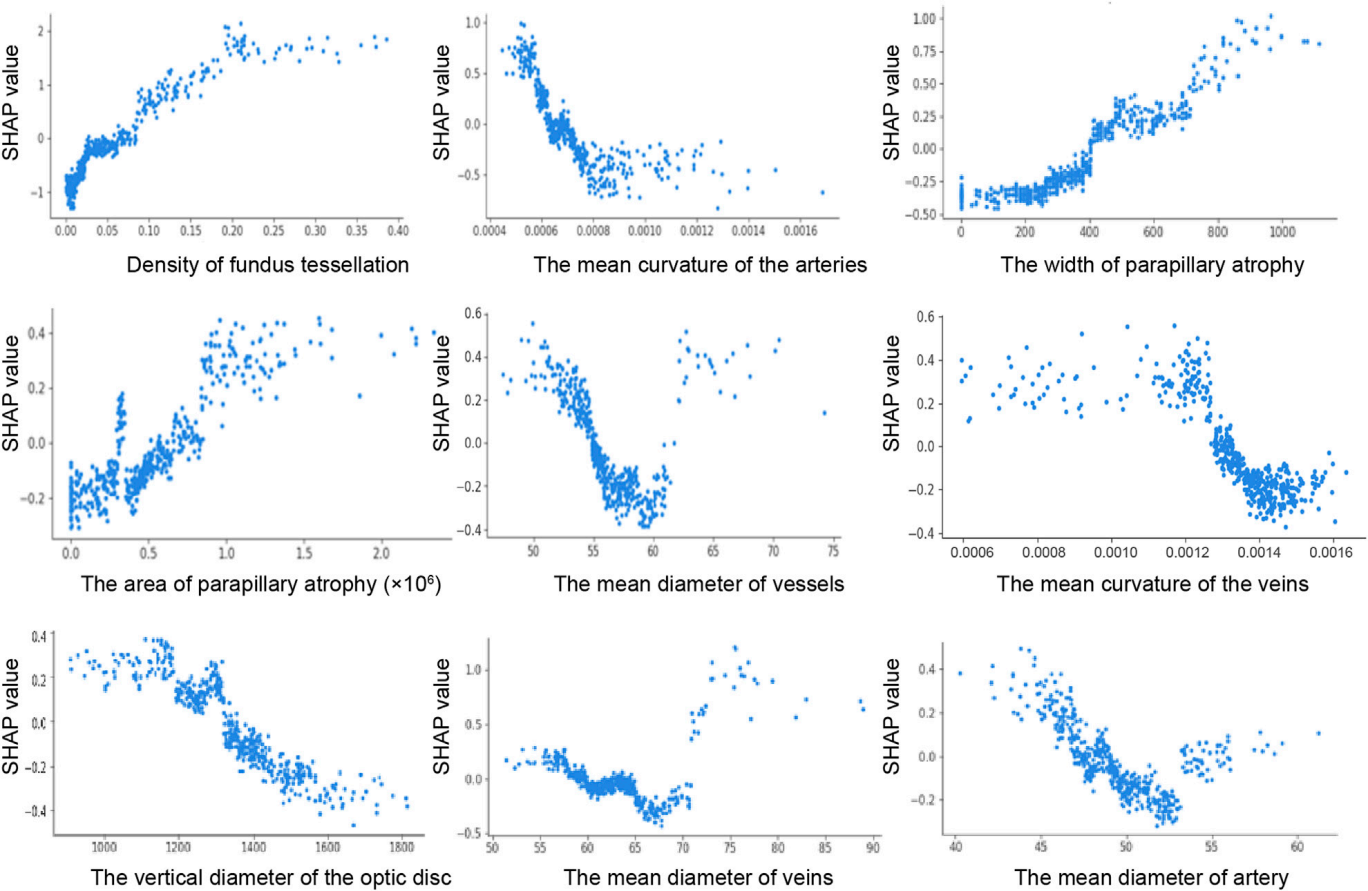
SHAP dependence plot. The y-axis shows the feature SHAP values. The SHAP values for specific features that exceed 0 represent an increased possibility of HM. The x-axis shows the feature ranges.

The SHAP dependence plot demonstrates the effect of a single feature on the output of the XGBoost model. We visualize the SHAP values of the nine features which appeared consistently in each iteration and analyze the relationships between them (Figure 3). When the SHAP value of each feature exceeds zero, this indicates an increased risk of HM. The tessellated density value greater than 0.025, width of parapapillary atrophy greater than 400 um, parapapillary atrophy area greater than 0.6 × 10^6^ um^2^ were associated with an increased risk of HM. The mean curvature of the arteries value over 0.00063, diameter of vessels over 55.2 um, curvature of the veins over 0.00128, vertical diameter of the optic disc over 1320 um, diameter of veins over 58.5 um, and diameter of artery over 47.1 um was associated with a decreased risk of HM. These findings highlight the key retinal features that significantly influenced the prediction of HM.

## 4 Discussion

This study presents a novel approach of employing an explainable ML framework based on retinal imaging omics to investigate high myopia-related fundus structural parameters. Unlike previous studies that relied entirely on algorithmic results, our method improved overall understanding by capturing and interpreting the entire retinal structure. We identified and ranked 29 parameters associated with the prediction. Of these, fundus tessellation density emerged as the strongest predictor. Additionally, during validation, we found out the effect of nine consistently present features on the predicted results, including increased tessellation density, width of parapillary atrophy, area of parapillary atrophy, and reduced mean arterial curvature, vessel diameter, vein curvature, vertical optic disc diameter, vein diameter, and artery diameter. The method provides robust evidence for doctors to accurately identify and predict HM.

Based on the META-PM classification and previous research, tessellation is recognized as an early indicator of myopic maculopathy (Ohno-Matsui et al., 2021; Yii et al., 2024). Most studies have used qualitative or semi-quantitative approaches to examine the severity of myopia and the degree of tessellation (Yamashita et al., 2018; Yan et al., 2015). Some studies (Shao et al., 2021; Huang et al., 2025; Li et al., 2023) quantified tessellation density using AI image processing technology and found a correlation relationship with the SE. However, to our knowledge, this was the first quantitative ordering of the structure of the entire retina. We measured tessellation patterns by calculating the average choroidal exposure per unit area to determine the tessellation density value greater than 0.025 increased the risk of HM. This feature consistently comes out on top in five validations underscores its critical role in predicting.

Arterial tortuosity was identified as the second most influential predictive factor after tessellation density. The risk of HM was reduced when the mean arterial curvature value exceeded 0.00063. Reduced arterial curvature is associated with tissue hypoxia (Malek et al., 2014). Hypoxia-induced vascular endothelial growth factor plays a role in the curvature of retinal arteriovenous system (Malek et al., 2015). Extrinsic factors such as shear stress and mechanical pressure contribute to variations in tortuosity (Kalitzeos et al., 2013). In patients with HM, increased axial length exerts lateral mechanical forces on the eye, potentially stretching the retina and making smaller arteries more susceptible to reduced curvature (Witt et al., 2006). These findings are consistent with Lim et al. (2011) observations using computer-assisted methodologies, HM exhibit significantly lower arterial tortuosity compared to non-high myopic individuals (P < 0.001). Importantly, this study also highlights the previously overlooked role of venous tortuosity in model predictions, suggesting that clinical procedures should evaluate both arterial and venous tortuosity to better assess retinal health and disease progression. Identifying the association between retinal vascular geometry and myopic changes is a critical research priority. Although previous studies have suggested that refractive error is independent of retinal vessel caliber (Cheung et al., 2007a; Cheung et al., 2007b), we founded that diameter of vessels less than 55.2 um, veins less than 58.5 um,and artery less than 47.1 um were positively associated with an increased risk of HM. Unlike previous studies that calculated the central retinal arteriolar and venular equivalents by summing individual retinal vessel diameters (Pappelis and Jansonius, 2023), we determined the ratio of image pixel size to the actual distance using a deep learning-recognized region of interest. This method provides a more accurate representation of the retinal vascular system (Long et al., 2022). By quantifying the global retinal vascular geometry using this method, a more accurate representation of the overall vascular condition can be obtained.

In this study, we found that both the width and area of parapapillary atrophy significantly contributed to the prediction of the model. Typically, parapapillary atrophy is located horizontally on the temporal side, followed by the supratemporal and inferotemporal regions, and this distribution affects the measurement of the height of atrophy (Fang et al., 2018). In addition, the gamma region cannot be accurately identified because the border of the Brush’s membrane could not be detected by fundus photography. Notably, the vertical diameter of the optic disc emerged as a significant factor in each iteration, offering new insights for clinical application in HM.

To date, the analysis of myopic fundus characteristics has relied primarily on subjective evaluations (Hu et al., 2020). Despite the ability to manually label a few structures, the lack of objective quantification due to empirical differences and methodological limitations precludes the comprehensive assessment of complex retinal parameters (Sun J. et al., 2018; Guo et al., 2021). Thus, the relationship between these structures and the severity of myopia remains poorly understood. Artificial intelligence (AI) offers the potential to improve the identification and segmentation for these structures. Our study employed the SHAP-XGBoost to quantify the impact of retinal characteristics on HM. XGBoost, a versatile nonparametric model, provides superior accuracy over linear models. Due to its ability to handle heterogeneous and high-dimensional clinical data, and its robustness in dealing with missing values and complex variables of different categories, it is particularly suitable for the structured and multi-factor nature of ophthalmic datasets (Chen and Guestrin, 2016). This method has been applied in previous studies on long-term outcomes of refractive surgery (Kim et al., 2022) and prognosis prediction of ocular trauma (Meng et al., 2024). Although improving predictive accuracy is essential, enhancing model interpretability is equally important for clinical credibility and utility. SHAP summary and dependence plots clarify the contribution of each variable to the model, providing valuable insights for interpretability in AI-based predictions.

### 4.1 Study limitations

This study has several limitations. First, although the data were collected from a single center, the cross-validation method confirmed that the model demonstrated satisfactory performance. Second, the cohort comprised solely adult patients with myopia. Future studies should include analyses of individuals older than 45 years and adolescents with myopia. Finally, the main purpose of this study is to investigate the role of retinal characteristics in HM, and the SHAP analysis provided valuable insights regarding how various features influenced the prediction model, although the axial length was not fully collected before the operation.

## 5 Conclusion

This study effectively utilized explainable ML frameworks based on retinal imaging omics to rank the contributions of 29 retinal features. Tessellation density emerged as the most significant marker, and when its value was greater than 0.025, the risk of developing HM increased significantly. In addition, parapillary atrophy width and area were associated with increased risk of HM prediction, mean curvature of the arteries, diameter of vessels, curvature of the veins, vertical diameter of the optic disc, diameter of veins, and diameter of artery was associated with reduced risk predicted by HM. These findings can enhance clinician understanding and confidence in using ML for high myopia prediction, thus expand its potential and applicability in ophthalmology.

## Data Availability

The raw data supporting the conclusions of this article will be made available by the authors, without undue reservation.

## References

[B1] Baird P. N. Saw S. M. Lanca C. Guggenheim J. A. Smith III E. L. Zhou X. (2020). Myopia. Nat. Rev. Dis. Prim. 6 (1), 99. 10.1038/s41572-020-00231-4 33328468

[B2] Chen T. Guestrin C. (2016). “Xgboost: a scalable tree-boosting system,” in Proceedings of the 22nd ACM sigkdd international conference on knowledge discovery and data mining (San Francisco, CA, USA), 785–794.

[B3] Chen N. Zhu Z. Yang W. Wang Q. (2024). Progress in clinical research and applications of retinal vessel quantification technology based on fundus imaging. Front. Bioeng. Biotechnol. 12, 1329263. 10.3389/fbioe.2024.1329263 38456011 PMC10917897

[B4] Cheung N. Tong L. Tikellis G. Saw S. M. Mitchell P. Wang J. J. (2007a). Relationship of retinal vascular caliber with optic disc diameter in children. Invest Ophthalmol. Vis. Sci. 48 (11), 4945–4948. 10.1167/iovs.07-0472 17962443

[B5] Cheung N. Tikellis G. Saw S. M. Amirul Islam F. Mitchell P. Wang J. J. (2007b). Relationship of axial length and retinal vascular caliber in children. Am. J. Ophthalmol. 144 (5), 658–662.e1. 10.1016/j.ajo.2007.07.023 17869206

[B6] Conti A. Duggento A. Indovina I. Guerrisi M. Toschi N. (2021). Radiomics in breast cancer classification and prediction. Semin. Cancer Biol. 72, 238–250. 10.1016/j.semcancer.2020.04.002 32371013

[B8] Fang Y. Yokoi T. Nagaoka N. Shinohara K. Onishi Y. Ishida T. (2018). Progression of myopic maculopathy during 18-Year Follow-up. Ophthalmology 125 (6), 863–877. 10.1016/j.ophtha.2017.12.005 29371011

[B9] Guo X. Chen X. Li M. Li S. You R. Wang Y. (2021). Association between morphological characteristics of the optic disc and other anatomical features of the fundus in highly myopic eyes. Eur. J. Ophthalmol. 31 (5), 2329–2338. 10.1177/1120672120945901 32757632

[B10] Haarman A. E. G. Enthoven C. A. Tideman J. W. L. Tedja M. S. Verhoeven V. J. M. Klaver C. C. W. (2020). The complications of myopia: a review and meta-analysis. Invest Ophthalmol. Vis. Sci. 61 (4), 49. 10.1167/iovs.61.4.49 32347918 PMC7401976

[B11] Hu G. Chen Q. Xu X. Lv H. Du Y. Wang L. (2020). Morphological characteristics of the optic nerve head and choroidal thickness in high myopia. Invest Ophthalmol. Vis. Sci. 61 (4), 46. 10.1167/iovs.61.4.46 32343784 PMC7401971

[B12] Huang D. Lin X. Zhu H. Ling S. Dong Z. Ke X. (2025). The associations between myopia and fundus tessellation in school children: a comparative analysis of macular and peripapillary regions using deep learning. Transl. Vis. Sci. Technol. 14 (1), 4. 10.1167/tvst.14.1.4 39775798 PMC11721481

[B13] Jong M. Jonas J. B. Wolffsohn J. S. Berntsen D. A. Cho P. Clarkson-Townsend D. (2021). IMI 2021 yearly digest. Invest Ophthalmol. Vis. Sci. 62 (5), 7. 10.1167/iovs.62.5.7 33909031 PMC8088231

[B14] Kalitzeos A. A. Lip G. Y. Heitmar R. (2013). Retinal vessel tortuosity measures and their applications. Exp. Eye Res. 106, 40–46. 10.1016/j.exer.2012.10.015 23146682

[B15] Kim J. Ryu I. H. Kim J. K. Lee I. S. Kim H. K. Han E. (2022). Machine learning predicting myopic regression after corneal refractive surgery using preoperative data and fundus photography. Graefes Arch. Clin. Exp. Ophthalmol. 260 (11), 3701–3710. 10.1007/s00417-022-05738-y 35748936

[B16] Li T. Bo W. Hu C. Kang H. Liu H. Wang K. (2021). Applications of deep learning in fundus images: a review. Med. Image Anal. 69, 101971. 10.1016/j.media.2021.101971 33524824

[B17] Li R. Guo X. Zhang X. Lu X. Wu Q. Tian Q. (2023). Application of artificial intelligence to quantitative assessment of fundus tessellated density in young adults with different refractions. Ophthalmic Res. 66 (1), 706–716. 10.1159/000529639 36854278

[B18] Lim L. S. Cheung C. Y. Lin X. Mitchell P. Wong T. Y. Mei-Saw S. (2011). Influence of refractive error and axial length on retinal vessel geometric characteristics. Invest Ophthalmol. Vis. Sci. 52 (2), 669–678. 10.1167/iovs.10-6184 20847122

[B19] Long T. Xu Y. Zou H. Lu L. Yuan T. Dong Z. (2022). A generic pixel pitch calibration method for fundus camera *via* automated ROI extraction. Sensors (Basel) 22 (21), 8565. 10.3390/s22218565 36366262 PMC9653591

[B20] Malek J. Azar A. T. Tourki R. (2014). Impact of retinal vascular tortuosity on retinal circulation. Neural. comput. Appl. 26 (1), 25–40.

[B21] Malek J. Azar A. T. Tourki R. (2015). Impact of retinal vascular tortuosity on retinal circulation. Neural Comput. Applic 26, 25–40. 10.1007/s00521-014-1657-2

[B22] Meng X. Wang Q. Chen S. Zhang S. Yu J. Li H. (2024). An interpretable model predicts visual outcomes of no light perception eyes after open globe injury. Br. J. Ophthalmol. 108 (2), 285–293. 10.1136/bjo-2022-322753 36596662

[B23] Modjtahedi B. S. Abbott R. L. Fong D. S. Lum F. Tan D. Task Force on Myopia (2021). Reducing the global burden of myopia by delaying the onset of myopia and reducing myopic progression in children: the academy's task force on myopia. Ophthalmology 128 (6), 816–826. 10.1016/j.ophtha.2020.10.040 33388160

[B24] Ohno-Matsui K. Lai T. Y. Lai C. C. Cheung C. M. (2016). Updates of pathologic myopia. Prog. Retin Eye Res. 52, 156–187. 10.1016/j.preteyeres.2015.12.001 26769165

[B25] Ohno-Matsui K. Wu P. C. Yamashiro K. Vutipongsatorn K. Fang Y. Cheung C. M. G. (2021). IMI pathologic myopia. Invest Ophthalmol. Vis. Sci. 62 (5), 5. 10.1167/iovs.62.5.5 33909033 PMC8083114

[B26] Pappelis K. Jansonius N. M. (2023). Retinal vessel caliber measurement bias in fundus images in the presence of the central light reflex. Transl. Vis. Sci. Technol. 12 (7), 16. 10.1167/tvst.12.7.16 37450282 PMC10353742

[B27] Ruiz-Medrano J. Montero J. A. Flores-Moreno I. Arias L. García-Layana A. Ruiz-Moreno J. M. (2019). Myopic maculopathy: current status and proposal for a new classification and grading system (ATN). Prog. Retin Eye Res. 69, 80–115. 10.1016/j.preteyeres.2018.10.005 30391362

[B28] Salih A. Boscolo Galazzo I. Gkontra P. Lee A. M. Lekadir K. Raisi-Estabragh Z. (2023). Explainable artificial intelligence and cardiac imaging: toward more interpretable models. Circ. Cardiovasc Imaging 16 (4), e014519. 10.1161/circimaging.122.014519 37042240

[B29] Shao L. Zhang Q. L. Long T. F. Dong L. Zhang C. Da Zhou W. (2021). Quantitative assessment of fundus tessellated density and associated factors in fundus images using artificial intelligence. Transl. Vis. Sci. Technol. 10 (9), 23. 10.1167/tvst.10.9.23 34406340 PMC8383900

[B30] Shinojima A. Negishi K. Tsubota K. Kurihara T. (2022). Multiple factors causing myopia and the possible treatments: a mini review. Front. Public Health 10, 897600. 10.3389/fpubh.2022.897600 35619815 PMC9127355

[B31] Sun R. Limkin E. J. Vakalopoulou M. Dercle L. Champiat S. Han S. R. (2018). A radiomics approach to assess tumour-infiltrating CD8 cells and response to anti-PD-1 or anti-PD-L1 immunotherapy: an imaging biomarker, retrospective multicohort study. Lancet Oncol. 19 (9), 1180–1191. 10.1016/s1470-2045(18)30413-3 30120041

[B32] Sun J. Wang J. You R. Wang Y. (2018). Is the retinal vasculature related to β-Peripapillary atrophy in nonpathological high myopia? An optical coherence tomography angiography study in Chinese adults. J. Ophthalmol. 2018, 1–8. 10.1155/2018/7895238 30363989 PMC6186342

[B33] Sun Q. Chen Y. Liang C. Zhao Y. Lv X. Zou Y. (2021). Biologic pathways underlying prognostic radiomics phenotypes from paired MRI and RNA sequencing in glioblastoma. Radiology 301 (3), 654–663. 10.1148/radiol.2021203281 34519578

[B34] Verhoeven V. J. Wong K. T. Buitendijk G. H. Hofman A. Vingerling J. R. Klaver C. C. (2015). Visual consequences of refractive errors in the general population. Ophthalmology 122 (1), 101–109. 10.1016/j.ophtha.2014.07.030 25208857

[B35] von Elm E. Altman D. G. Egger M. Pocock S. J. Gøtzsche P. C. Vandenbroucke J. P. (2007). The strengthening the reporting of observational studies in epidemiology (STROBE) statement: guidelines for reporting observational studies. PLoS Med. 4 (10), e296. 10.1371/journal.pmed.0040296 17941714 PMC2020495

[B36] Witt N. Wong T. Y. Hughes A. D. Chaturvedi N. Klein B. E. Evans R. (2006). Abnormalities of retinal microvascular structure and risk of mortality from ischemic heart disease and stroke. Hypertension 47 (5), 975–981. 10.1161/01.hyp.0000216717.72048.6c 16585415

[B37] Xu L. Zhuang Y. Zhang G. Ma Y. Yuan J. Tu C. (2021). Design, methodology, and baseline of whole city-million scale children and adolescents myopia survey (CAMS) in wenzhou, China. Eye Vis. (Lond). 8 (1), 31. 10.1186/s40662-021-00255-1 34407890 PMC8373605

[B38] Yamashita T. Iwase A. Kii Y. Sakai H. Terasaki H. Sakamoto T. (2018). Location of ocular tessellations in Japanese: population-based kumejima study. Invest Ophthalmol. Vis. Sci. 59 (12), 4963–4967. 10.1167/iovs.18-25007 30326064

[B39] Yan Y. N. Wang Y. X. Xu L. Xu J. Wei W. B. Jonas J. B. (2015). Fundus tessellation: prevalence and associated factors. Ophthalmology 122 (9), 1873–1880. 10.1016/j.ophtha.2015.05.031 26119000

[B40] Yii F. Nguyen L. Strang N. Bernabeu M. O. Tatham A. J. MacGillivray T. (2024). Factors associated with pathologic myopia onset and progression: a systematic review and meta-analysis. Ophthalmic Physiol. Opt. 44 (5), 963–976. 10.1111/opo.13312 38563652 PMC12862026

[B7] Yu Y.D. He Z. Ouyang J. Tan Y. Chen Y. Gu Y. (2021). Magnetic resonance imaging radiomics predicts preoperative axillary lymph node metastasis to support surgical decisions and is associated with tumor microenvironment in invasive breast cancer: a machine learning, multicenter study. Ebiomedicine 69, 103460. 10.1016/j.ebiom.2021.103460 34233259 PMC8261009

[B41] Zhang H. Zhang H. Jiang M. Li J. Li J. Zhou H. (2025). Radiomics in ophthalmology: a systematic review. Eur. Radiol. 35 (1), 542–557. 10.1007/s00330-024-10911-4 39033472

