# Supplementary material for: Retinal features as predictive indicators for high myopia: insights from explainable multi-machine learning models
Source: Front Bioeng Biotechnol. 2025 Oct 13;13:1609639. doi: 10.3389/fbioe.2025.1609639 (PMC12555007; doi:10.3389/fbioe.2025.1609639)
Supplement: Supplementary file 1 [file Supplementaryfile1.docx]

Document S1 Feature recognition and quantification

In this study, we employed the retinal image intelligent analysis software [1-3]to identify, segment, and quantify the optic disc, optic cup, parapapillary atrophy, tessellated density, and retinal vascular structures in the following steps. This software employed deep learning-based semantic segmentation networks tailored to distinct fundus structures. The annotated samples were randomly split into training: validation: test sets (8:1:1). The model was trained on the training set, with loss values calculated on the validation set. Parameters were optimized by monitoring the loss values until they stabilized (ceased to decrease) in both training and validation sets, yielding the final segmentation model. During training, data augmentation (primarily random horizontal/vertical flipping) was applied to enhance generalization, using pixel-wise cross-entropy loss as the objective function. Evaluation metrics were computed on the test set. All models achieved accuracy and specificity ≥0.95, sensitivity ≥0.85, and intersection-over-union >0.7.

**1. Image preprocessing**

To remove the interference of noisy regions, reduce the variability between images, and improve the sharpness of feature edges, each image was first preprocessed, including extraction, denoising, normalization, and enhancement operations [4-6].

**2. Extraction of optic disc and cup**

The optic disc region was located using Single Shot Detection (SSD), and a rectangular detection box for the optic disc was obtained. Within the detection box, a polar coordinate transformation was applied centered on the optic disc's center point. This is followed by edge detection to identify the optic disc's boundary. Subsequently, a reverse polar transformation was performed to obtain the optic disc's boundary in Cartesian coordinates, achieving segmentation of the optic disc region [4]. The image of the optic disk region within the rectangular detection frame was then fed into the ResNet-101-UNet model to obtain the edge coordinates and segmentation image of the optic cup [1]. The resulting optic disk and optic cup are subsequently utilized as a basis for quantifying their respective feature parameters.

| 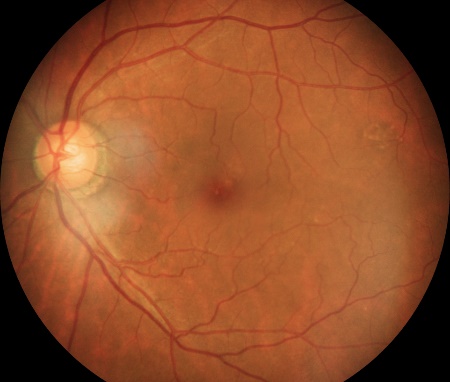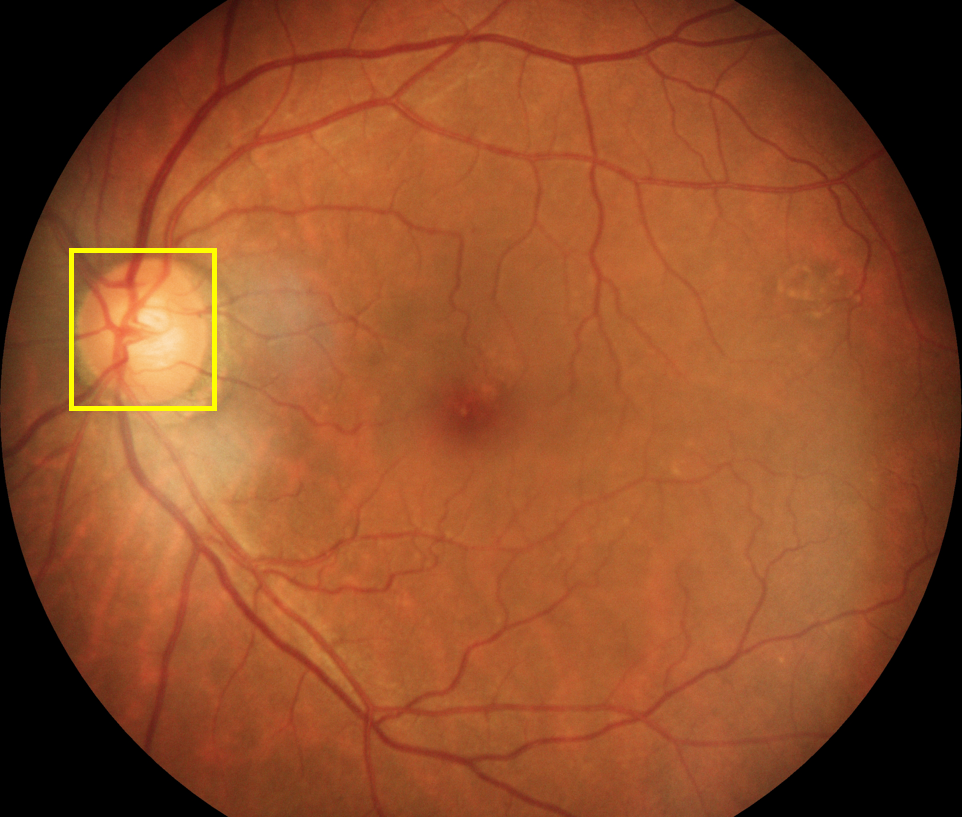 |  |
| --- | --- |
| Original image | Optic disc localization |
| 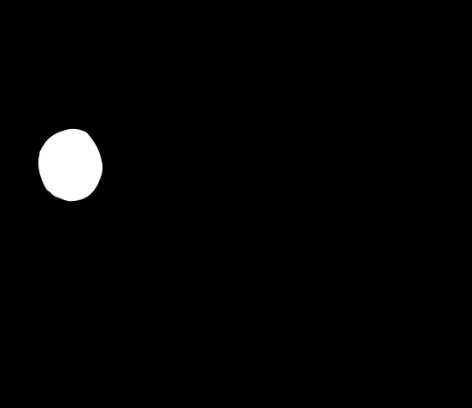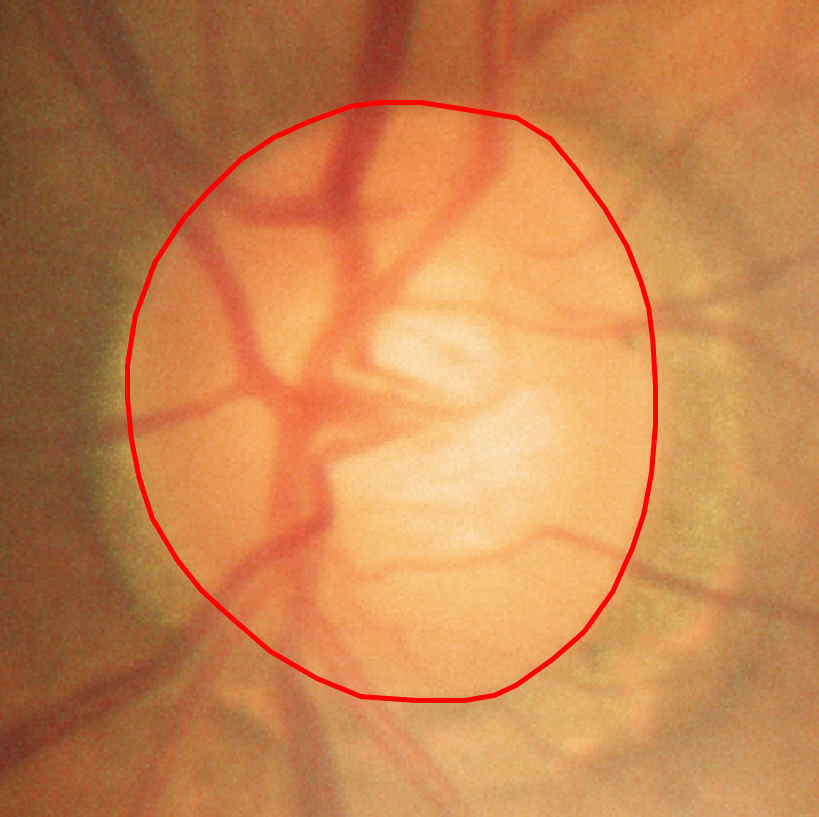 |  |
| Optic disc segmentation | Results of segmentation |
|  | 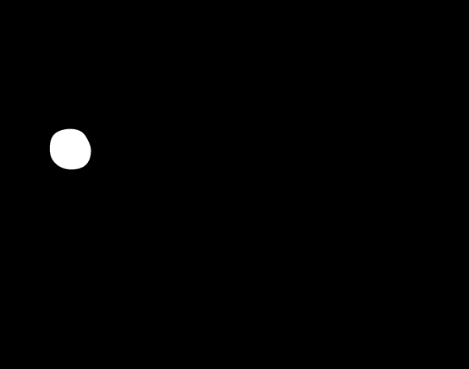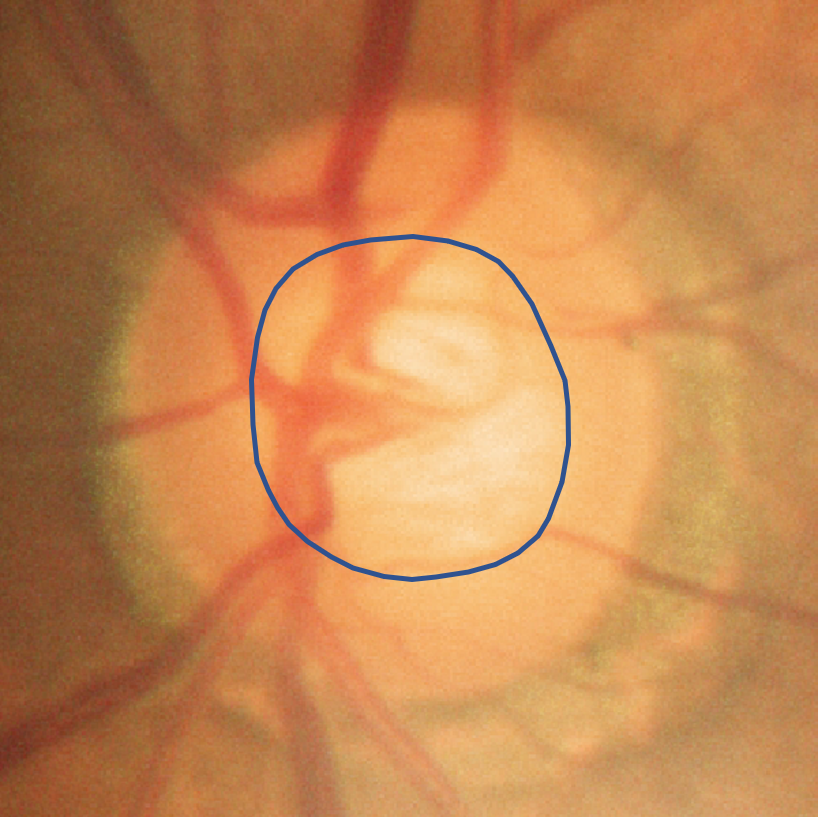 |
| Optic cup segmentation | Results of segmentation |

**3. Extraction of parapapillary atrophy**

A deep learning semantic segmentation network, ResNet-101-UNet, was used to construct a model for segmenting parapapillary atrophy, with training samples annotated and validated by two experienced physicians. Subsequently, the feature parameters of the atrophic arc were computed based on the segmented region. In this study, since we were conducting fine segmentation of the structural features of the fundus (such as blood vessels, leopard spots, etc.), to ensure the accuracy of the samples, we referred to the segmentation labeling methods used by He et al. [2] and Shi et al. [9]. Two experienced doctors were involved. One of them was responsible for the initial marking, while the other reviewed and corrected the markings after they were completed. The image after the second doctor's review and correction was used as the final sample. During the process, we ensured the accuracy of the samples through double-person annotation, and the doctors involved in the annotation had more than 5 years of working experience. The second doctor who reviewed and corrected had even more extensive clinical work experience of more than 8 years. Therefore, we used the sample modified and reviewed by the second doctor as the final sample.

| 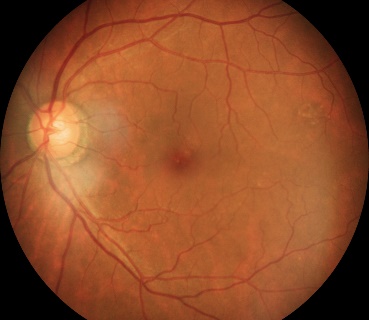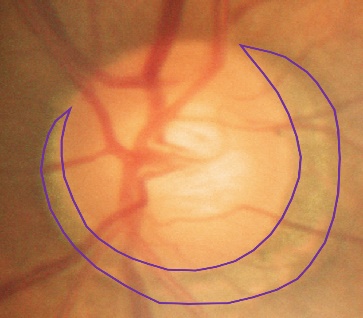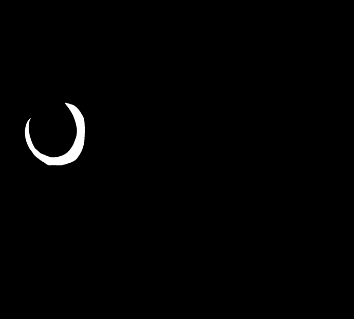 |  |  |
| --- | --- | --- |
| Original image | Parapapillary atrophy segmentation | Results of segmentation |

**4. Extraction of** **fundus tessellation**

The semantic segmentation network (Resnet FCN) was used to train the model. First extract high-level features of images, and then deconvolution to obtain the segmentation area, output the leopard spot confidence map, and obtained the confidence probability of each pixel on the fundus belongs to exposed choroid, and finally the exposed choroid of fundus was obtained through threshold segmentation. The tessellation density was calculated to describe the distribution of fundus tessellation based on the recognition results [6-8].

|  | 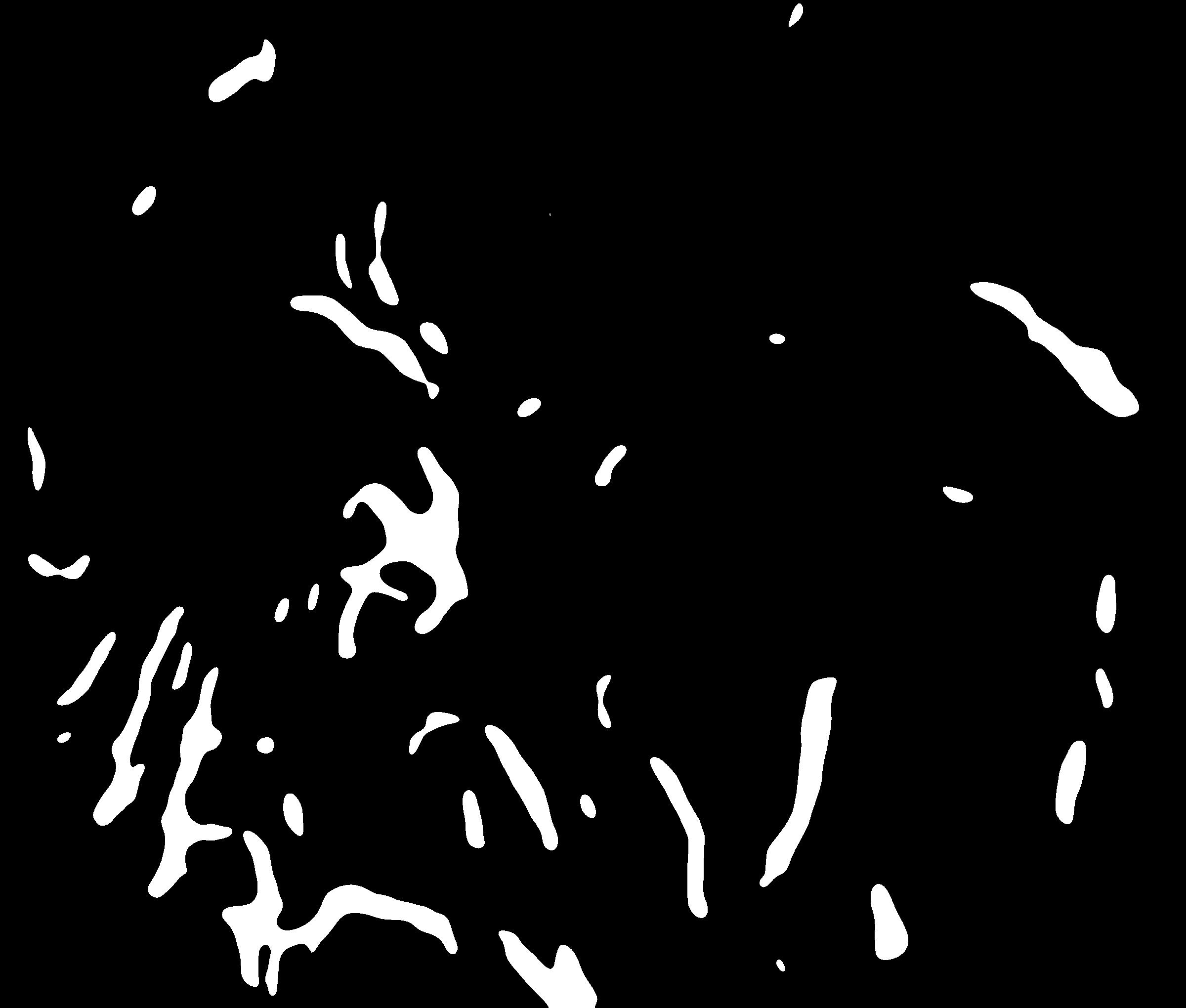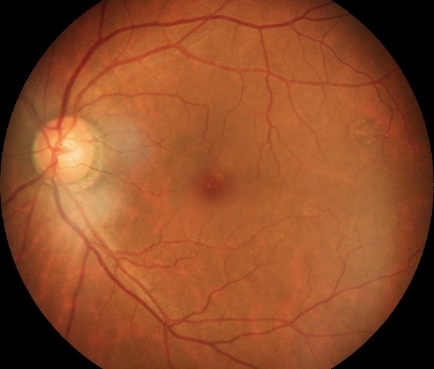 |
| --- | --- |
| Original image | fundus tessellation segmentation |

**5. Extraction of vascular**

The pre-trained segmentation model was used to obtain the vascular segmentation results [9]. Arteries and veins are identified based on the color, brightness and texture features of arteries and veins on the image, as well as vascular links and topological relationships [1,3], respectively. Morphological erosion operation was performed on the segmented artery and vein vessels to obtain the vessel center line. The distance between the two points was the vessel diameter corresponding to the point on the center line. The curvature corresponding to the point on the center line was taken as the vessel tortuosity corresponding to the point.

|  | 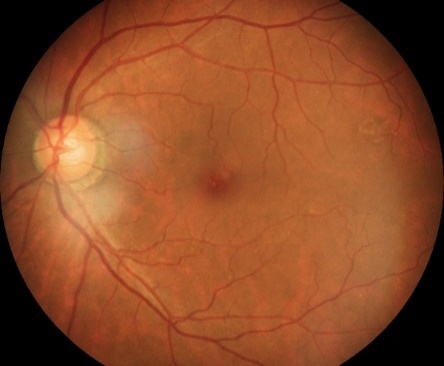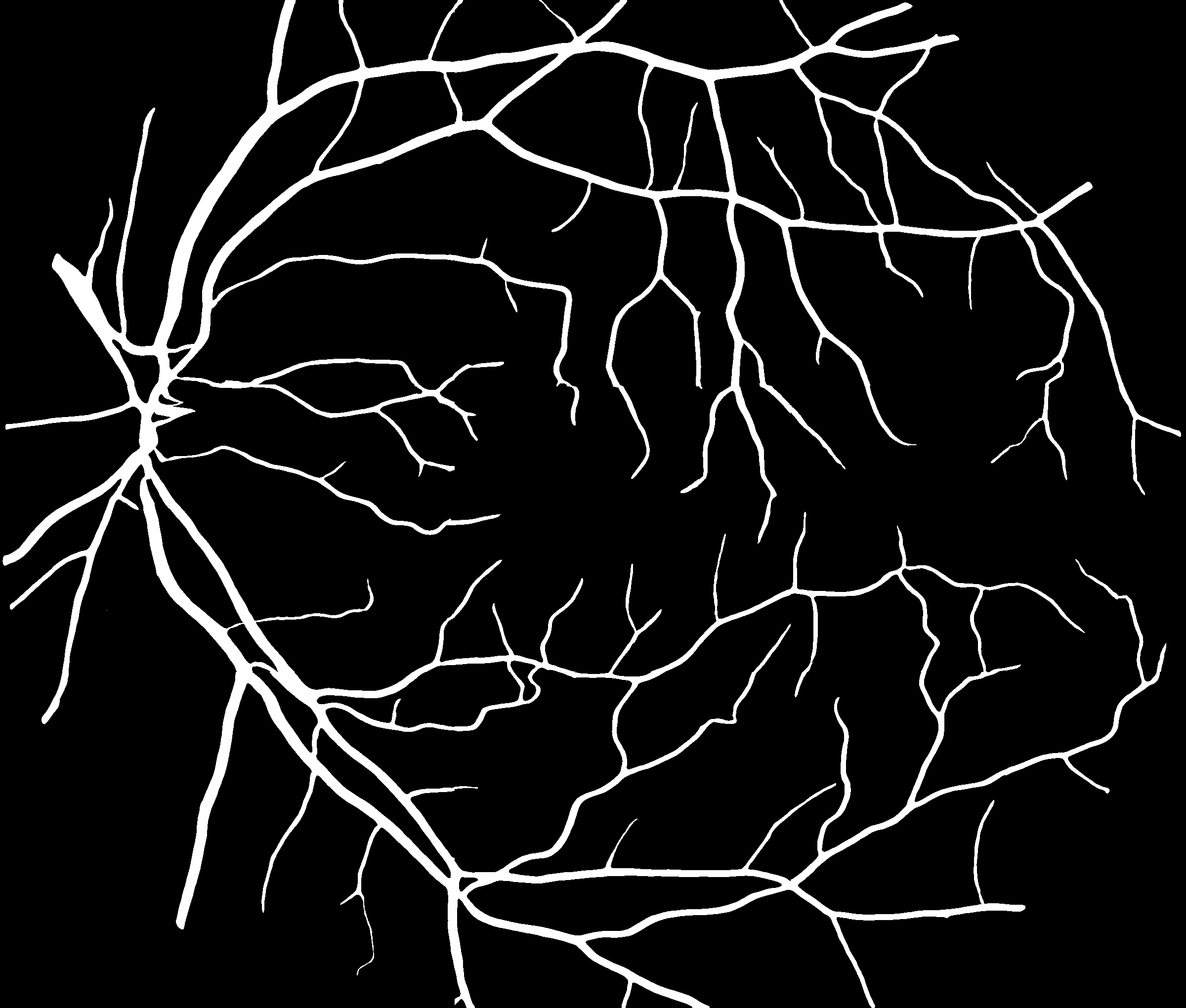 |
| --- | --- |
| Original image | Identification of vascular |
| 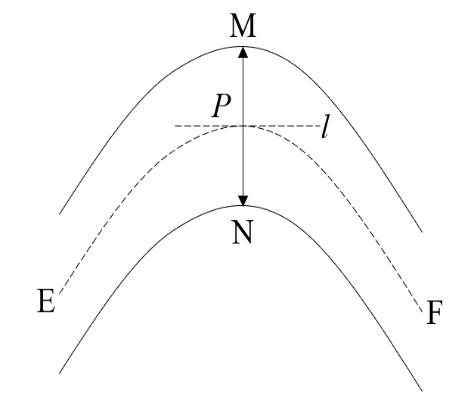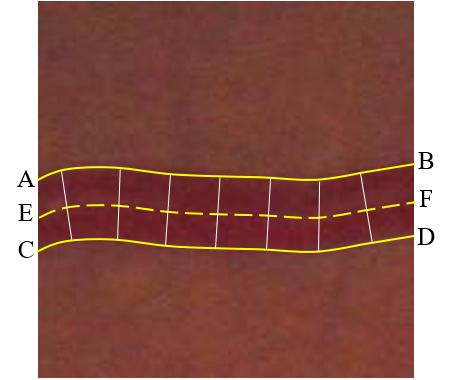 |  |
| Calculation of tortuosity | Calculation of vessel diameter |

**6. Key Indicators and Definitions:**

| Optic disc area | The area of the optic disc obtained by segmentation |
| --- | --- |
| Horizontal diameter of the optic disc | The diameter in the horizontal direction of the minimal circumscribed circle of the optic disc |
| Vertical diameter of the optic disc | The diameter in the vertical direction of the minimal circumscribed circle of the optic disc |
| Optic cup area | The area of the optic cup obtained by segmentation |
| Horizontal diameter of the optic cup | The diameter in the horizontal direction of the minimal circumscribed circle of the optic cup |
| Vertical diameter of the optic cup | The diameter in the vertical direction of the minimal circumscribed circle of the optic cup |
| Cup-to-disc area ratio | The area ratio of the optic cup to the optic disc |
| Horizontal cup-to-disc ratio | Ratio of the horizontal diameter of the optic cup to the horizontal diameter of the optic disc |
| Vertical cup-to-disc ratio | The ratio of the vertical diameter of the optic cup to the vertical diameter of the optic disc |
| Parapapillary atrophy area | The area of the Parapapillary atrophy |
| Height of parapapillary atrophy | The length of the minimal external matrix in the vertical direction of the parapapillary atrophy |
| Width of parapapillary atrophy | The distance between the point on the central line of the parapapillary atrophy and the shortest segment of the intersection of the two arc-shaped edges |
| Parapapillary atrophy-to-optical disc area ratio | The area ratio of the parapapillary atrophy to the optic disc |
| Width of parapapillary atrophy-to-horizontal diameter of the optic disc ratio | The ratio of the width of the parapapillary atrophy to the horizontal diameter of the optic disc |
| Height of parapapillary atrophy-to-vertical diameter of the optic disc ratio | The ratio of the height of the parapapillary atrophy to the vertical diameter of the optic disc |
| Width of inferior rim | Distance between the boundary of the optic disc and the optic cup on the inferior side in the vertical direction of the optic disc |
| Width of superior rim | Distance between the upper side of the optic disc and the optic cup boundary in the vertical direction of the optic disc |
| Width of nasal rim | Distance between the nasal side of the optic disc and the optic cup boundary in the horizontal direction of the optic disc |
| Width of temporal rim | Distance between the temporal disc and cup boundaries in the horizontal direction of the optic disc |
| Fractal dimension | A measure of vascular irregularity that reflects the complexity of retinal vascular morphology. The metric is calculated based on the counting box dimension method |
| Vessel density | The area of retinal blood vessels per unit area. |
| Vascular tortuosity | The mean of the curvature of the points on the center line of all vessels |
| Arterial tortuosity | The mean of the curvature of the points on the center line of all arteries |
| Venous tortuosity | The mean of the curvature of the points on the center line of all veins |
| Mean vessel diameter | The mean vessel diameter corresponding to the points on the center line of all vessels |
| Mean arterial diameter | The mean vessel diameter corresponding to the points on the center line of all arteries |
| Mean venous diameter | The mean vessel diameter corresponding to the points on the center line of all veins |
| Arterial-to-venous ratio | The ratio of the mean arterial vessel diameter to the mean venous vessel diameter |
| Tessellated density | The area of choroidal exposure per unit area |

[1] Liu F, Yu XH, Wang YC, Cao M, Xie LF, Liu J, Liu LL. Quantitative analysis of optic disc changes in school-age children with ametropia based on artificial intelligence. Int J Ophthalmol 2023;16(11):1727-1733.

[2] He HL, Liu YX, Chen XY, et al. Fundus Tessellated Density of Pathologic Myopia. Asia Pac J Ophthalmol (Phila). 2023;12(6):604-613.

[3] Zhao. L, Chen. Y, Jiang. B, et al. Correlation study of retinal vascular morphological parameters with ischemic stroke [J]. Chinese Journal of Ocular Fundus Diseases, 2022, 38(12): 1001-1005.

[4] Long, T.; Xu, Y.; Zou, H.; Lu, L.; Yuan, T.; Dong, Z.; Dong, J.; Ke, X.; Ling, S.; Ma, Y. A Generic Pixel Pitch Calibration Method for Fundus Camera via Automated ROI Extraction. Sensors 2022, 22, 8565.

[5] Xu Y, Wang Y, Liu B, et al. The diagnostic accuracy of an intelligent and automated fundus disease image assessment system with lesion quantitative function (SmartEye) in diabetic patients. BMC Ophthalmol. 2019;19(1):184.

[6] Shao L, Zhang QL, Long TF, Dong L, Zhang C, Da Zhou W, Wang YX, Wei WB. Quantitative assessment of fundus tessellated density and associated factors in fundus images using artificial intelligence. Transl Vis Sci Technol. 2021;10(9):23.

[7] Guo X, Li R, Lu X, et al. Quantization of Optic Disc Characteristics in Young Adults Based on Artificial Intelligence. Curr Eye Res. 2023;48(11):1068-1077.

[8] Huang D, Qian Y, Yan Q, et al. Prevalence of Fundus Tessellation and Its Screening Based on Artificial Intelligence in Chinese Children: the Nanjing Eye Study. Ophthalmol Ther. 2023;10.1007

[9] Shi XH, Dong L, Zhang RH, et al. Relationships between quantitative retinal microvascular characteristics and cognitive function based on automated artificial intelligence measurements. Front Cell Dev Biol. 2023;11:1174984.
