# Supplementary material for: Retinal features as predictive indicators for high myopia: insights from explainable multi-machine learning models
Source: Front Bioeng Biotechnol. 2025 Oct 13;13:1609639. doi: 10.3389/fbioe.2025.1609639 (PMC12555007; doi:10.3389/fbioe.2025.1609639)
Supplement: Supplementary file 2 [file Supplementaryfile2.docx]

In the construction of the XGBoost model, we systematically optimized hyperparameters including learning rate, maximum depth, subsample ratio, and column sample ratio through grid search combined with Bayesian optimization. The optimal parameter combination was selected using 5-fold cross-validation. During model training, an early stopping mechanism was implemented to terminate training when the validation set's AUC showed no improvement for 20 consecutive iterations, thereby mitigating overfitting risks.

Regarding data normalization, all input features were standardized using z-score normalization (mean = 0, standard deviation = 1) to eliminate dimensional differences. Non-continuous variables (e.g., gender) were converted into numerical features via One-Hot encoding. Stratified sampling was employed for validation to ensure consistent proportions of high myopia and non-high myopia groups (1191:1790) between the training and validation sets, preventing data distribution bias.

For class imbalance handling, given the imbalance between high myopia samples (40%) and non-high myopia samples (60%), we adopted a weighted loss function by setting the scale_pos_weight parameter in XGBoost to enhance the model's sensitivity to the minority class (i.e., high myopia group). During 5-fold cross-validation, the sample ratio of high myopia to non-high myopia was maintained consistent across each fold to avoid validation bias.

To address potential overfitting of the XGBoost model, we enhanced generalization ability through the following measures: L1 (lambda) and L2 (alpha) regularization terms were incorporated into the model to prevent overfitting caused by complex feature combinations.
